# Social Interventions to Prevent Heat-Related Mortality in the Older Adult in Rome, Italy: A Quasi-Experimental Study

**DOI:** 10.3390/ijerph15040715

**Published:** 2018-04-11

**Authors:** Giuseppe Liotta, Maria Chiara Inzerilli, Leonardo Palombi, Olga Madaro, Stefano Orlando, Paola Scarcella, Daniela Betti, Maria Cristina Marazzi

**Affiliations:** 1 Department of Biomedicine and Prevention, University of Rome “Tor Vergata”—Via Montpellier 1, 00173 Roma, Italy; leonardo.palombi@gmail.com (L.P.); stefano.orlando@dreameurope.org (S.O.); paola.scarcella@gmail.com (P.S.); 2 Community of Sant’Egidio, “Long Live the Elderly” Program—Via San Gallicano 25, 00153 Roma, Italy; chiarainzerilli@gmail.com (M.C.I.); olga.madaro@tin.it or soli.no@santegidio.org (O.M.); betti.daniela@gmail.com (D.B.); 3 Direction of Care Services, Municipality of Rome—Viale Manzoni 16, 00185 Roma, Italy; 4 Local Health Unit “Roma 3”—Via Casal Bernocchi 73, 00125 Roma, Italy; 5 Department of Human Studies, LUMSA University—Via Della Traspontina 21, 00193 Roma, Italy; mcmarazzi@gmail.com

**Keywords:** heat-related mortality, older adults, social care program, urban environment

## Abstract

This study focuses on the impact of a program aimed at reducing heat-related mortality among older adults residing in central Rome by counteracting social isolation. The mortality of citizens over the age of 75 living in three Urban Areas (UAs) located in central Rome is compared with that of the residents of four adjacent UAs during the summer of 2015. The data, broken down by UA, were provided by the Statistical Office of the Municipality of Rome, which gathers them on a routine basis. During the summer of 2015, 167 deaths were recorded in those UAs in which the Long Live the Elderly (LLE) program was active and 169 in those in which it was not, implying cumulative mortality rates of 25‰ (SD ± 1.4; Cl 95%: 23–29) and 29‰ (SD ± 6.7; Cl 95%: 17–43), respectively. Relative to the summer of 2014, the increase of deaths during the summer of 2015 was greater in UAs in which the LLE program had not been implemented (+97.3% vs. +48.8%). In conclusion, the paper shows the impact of a community-based active monitoring program, focused on strengthening individual relationship networks and the social capital of the community, on mortality in those over 75 during heat waves.

## 1. Introduction

Heat-related mortality is a growing public health concern worldwide because of the increased number of older adults highly susceptible to the effects of heat. In fact, heat-related mortality amongst those over 75 is becoming a major issue because of the high prevalence of frail individuals, who show a higher risk of death as a consequence of psycho-physical impairment and/or lack of socio-economic resources [1,2,3]. In this context, the heat waves that hit Southern Europe during the summers of 2003 and 2015 were the most relevant episodes in causing an increase of deaths in older adults [4,5,6,7,8,9,10,11,12]. Prevention programs implemented after the summer of 2003 seem to have had a slight impact on mortality during the milder summers, while no impact has been noted during the hottest summers [13]. Apart from age, gender is one of the main factors related to mortality during heat waves, with a higher impact being recorded for older women than for older men, though this differential seems to be reduced in the over 75 population [14,15].

Several factors prevent an accurate assessment of the reduction in the impact of heat waves following the implementation of heat-related mortality prevention programs; in practice, most prevention programs are aimed at the general population of a city (or even a country), so that most studies are based on historical research rather than on a comparison of those sections of a population that are included or not included in prevention activities [16,17]. Moreover, a comparison between different areas, even within the same city, can only be approximate because of locally different climatic conditions (temperature, pollution, humidity and other variables), which can affect the impact of heat waves on health [18,19]. Over the years, however, a reduction in heat-related mortality has been noted, probably arising from interventions, such as campaigns on healthy life-styles and on adaptation strategies during heat waves, which were aimed at the population at large [12,13].

The Long Live the Elderly (LLE) program has been running for 13 years in some Urban Areas (UAs) of central Rome [20]. It was started in 2004 with the purpose of mitigating the effects of extreme atmospheric phenomenon such as cold and/or hot spells, after the severe heat wave of 2003, which especially hit older adults in Italy and throughout Europe. The program is aimed at those over 75 and focused on strengthening social relations around isolated and/or sick individuals, in order to increase social capital at both individual and community levels. Social capital could be defined as resources embedded in a social structure that are accessed and/or mobilized in purposeful actions [21,22]. In fact, the role played by social isolation is well known as a risk factor for the occurrence of heat-related deaths, especially in people who are suffering from physical impairment due to various diseases [23,24,25]. At the same time, the presence of a supportive social environment has been associated with a reduced mortality [26,27,28]. To our knowledge, however, the impact on heat-related mortality of strengthening a weak social relationship network or even constructing such a network around socially-isolated individuals is not known. In other words, the question is whether a program aimed at identifying isolated and/or sick individuals, and then supporting them with social interventions, shows the same positive impact on the mortality due to extreme climatic episodes as a “natural” network of relationships. The purpose of this study is to assess the impact of the LLE program by comparing UAs in which the program is active with those within the same administrative district of the city where it is not.

## 2. Methods

### 2.1. Population and Settings

**Figure 1:**
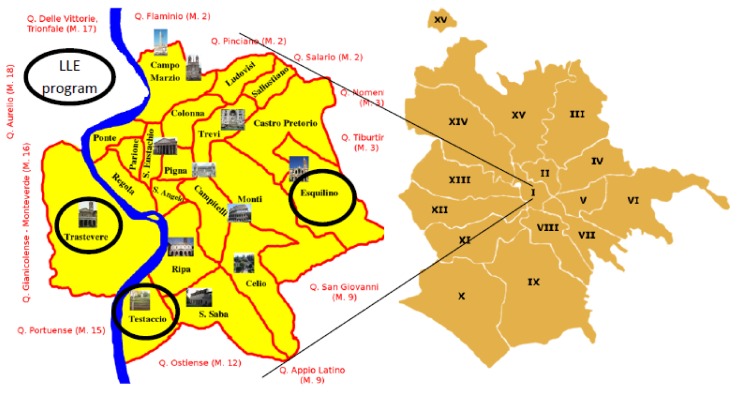
Rome, 1st administrative district (2015). LLE, Long Live the Elderly program.

The study design is a quasi-experimental retrospective cohort study. The impact of the intervention program has been assessed by comparing the death rate between two populations living in the first administrative urban district of Rome (Figure 1, Table 1). The two populations were divided between cases (6483 people residing in the UAs served by the LLE program) and controls (5724 people residing in the remaining UAs of the first district), comparable for age and gender.

### 2.2. Definition of Heat Waves

The definition of heat wave made by the Heat Health Watch Warning Systems (HHWWS), and implemented in Italy at the national level by the national prevention program of heat-related risk effects on population health, results from a combination of two factors: the air-mass model (a combination of air temperature, dew point temperature, atmospheric pressure, wind speed and direction) with the maximum apparent Temperature (*T*_appmax_), which is a discomfort index based on air dew point temperature, calculated using the following formula [29,30]:

*T*_apparent_ = −2.653 + 0.994(*T*_air_) + 0.0153(*T*_dew point_)

Both models have been matched with the retrospective analysis of mortality data [29,30,31] to define the thresholds associated with negative impacts on the population; Level 0 is made up by the values of both factors not associated with an increase of mortality. Level 1 is associated with an increase of mortality lower than 20% of the standard mortality for that period of the year. Level 2 is defined as the *T*_appmax_ values associated with an increase of mortality higher than 20% or to an air-mass model associated with any increase of mortality. Level 3 is the persistence of Level 2 for three or more days [31]. The Civil Protection Department, which, together with the Italian Ministry of Health, implements the national program for the prevention of heat effects on health, releases alerts aimed at the general population living in 34 cities with more than 200,000 inhabitants; special attention is given to messages aimed at older adults. The program is modelled on national campaigns on life-style and on the identification of susceptible subgroups (mainly older adults requiring higher care needs, identified through clinical and administrative data managed by the local administrative bodies). Such subgroups are the target of local prevention activities. When a heat wave is declared, based on the information gathered through the HHWWS, GPs are supposed to telephone their patients, who would already have been included in the prevention plan, and visit them at home in if necessary. During the summer of 2015, 23 alerts (Level 2 or more) were recorded in the city of Rome, while during the years 2014 and 2016, there were 2 and 5 alerts, respectively [13].

### 2.3. The Intervention

In 2004, the LLE program, aimed at the population over 75, was implemented in the “Testaccio” and “Trastevere” UAs of Rome and in 2011 in that of “Esquilino”; the three main goals were: (a) to contact all those over 75 for the purpose of offering them a periodic assessment of their social and health needs, health promotion campaigns (i.e., “Tips for hot weather”), assistance in handling bureaucratic matters or seeking formal or informal care and to provide details of the office, active from 8 a.m.–5 p.m., Monday–Friday, to contact if necessary; (b) to strengthen the community network around sick and/or socially-isolated individuals by involving people living or working near them in volunteer care actions; and (c) to increase the awareness of the community about the needs of older adults.

The LLE program promotes a proactive approach to reach the whole targeted population, so as to avoid some individuals being neglected because of a lack of awareness of their care needs. Based on a list provided by the municipality, all those over 75 receive a letter and then a phone call to obtain their consent to be part of the program; the percentage of refusal is lower than 2%. If the client accepts, then a multidimensional evaluation of their care needs is performed, and the service begins. According to the risk of a negative event, as assessed by the multidimensional evaluation, an individual care plan is drafted (including the services needed, even if the program will not provide these services, but will facilitate their provision to the client), and the client is included on the list for periodical phone calls: the higher the risk of negative events, the more frequently the person will be called, with a maximum frequency of once every two weeks, unless specific actions need to be taken. The activities of the program are intensified when a heat wave occurs: all those over 75 assisted by the program are traced by phone, and if necessary, the staff intervene with a home visit, bringing food and/or medicines as necessary, or involving the client’s network of relationships. It must be taken into account that a part of the population took holidays out of the city and that some of the declared residents were not in fact living at their official home addresses. Throughout the year, the operators act as a liaison between the older adults assisted by the program and the community, in order to increase the social capital of both. Healthy 75 year olds, however, are also contacted at least three times a year and during climatic emergencies, in order to monitor their situation.

### 2.4. Data Analysis

This analysis is focused on the death rate during the summer of 2015 and is based on data collected through standard procedures and periodically published by the Statistical Office of the Municipality of Rome (total population and total number of deaths by month, from June 2014–December 2016, in Rome’s first administrative district). However, a special dataset for this study was provided to the authors by the Statistical Office of the Municipality (see the Supplementary Materials—file_data.xls): published data are usually aggregated by administrative district, but in this case, data were broken down according to the UAs that made up the first administrative district. Because of the limited and homogeneous geographical areas taken into consideration [18], it was assumed that climatic conditions were uniform in central Rome. No account was therefore taken of meteorological variations between the UAs. Moreover, the analysis does not focus on the impact of a single heat wave or on the accumulation of several heat waves, but on the whole summer period. The assumption is that the impact of heat waves on those sections of the population showing a high prevalence of chronic diseases, like older adults, could persist longer than at any other age, as is often the case with other diseases affecting older adults. This is one of the reasons resulting in hospitalizations, the duration of which is 40% greater for those over 75 than for the 45–64 age group, independent of the disease causing admission [32]. Moreover, illness caused by or worsened by weather conditions could have serious consequences, including death, several days after the end of the heat wave; in the case of a series of heat waves, it would be very difficult to differentiate between the direct effect of climatic conditions in summer and indirect effects, i.e., general poor health, mediating the impact of climate on an individual’s health status in old age. All the deaths recorded from June–September 2015 are compared to those registered in the same period of 2014, in order to allow a homogeneous framework. Ideally, the period before and after an event should have been longer, in order to set up an average death rate as a baseline against which to measure the 2015 death rate variations. Unfortunately, the statistical office of the municipality could not supply death rates broken down by UA before May 2014.

Conceptual issues are raised by the variation of death rates where services have been implemented to take care of segments of the population, like those over 75, with a higher risk of death [13]. In fact, the analysis should take into account the potential protective effect of the program, as well as the potential mortality displacement effect due to the pre-summer weather condition. The winter of 2014–2015 saw an increase of mortality, mainly amongst those over 80 [10], with a potential impact on the size of the pool of individuals susceptible to extreme climate events in the following months. These factors have been taken into consideration by including in the analysis the pre-summer 2015 mortality (October 2014–May 2015) as a control factor (Table 2 and Table 3). Moreover, to give a comprehensive picture of the mortality trend, the death rate observed in October-November 2015 and the variation with respect to the summer 2015 mortality are shown (Table 2).

Age-specific death rates of those over 75 and the percentage variations of age-specific death rates for summer 2015 have been compared with those of summer 2014 in order to assess the impact on mortality of the greater number of heat alerts in 2015. A list of variables (see Supplementary Materials, Table S1) has been matched with the 2014–2016 death rates in order to select the ones showing a statistically-significant association to be included in the final multivariable model. Because of the well-known impact of socio-economic resources on mortality, several variables have been tested to include in the analysis as a proxy of the citizens’ socio-economic status; among the data available per urban areas, the property tax valuation has been chosen because of its relationship with mortality. Differences between the average mortality rates in the UAs where the LLE program was active and those in which it was not have been assessed by non-parametric tests and univariate regression. A multivariable linear regression model has been applied to check differences in mortality variations between UAs adjusted to the pre-summer 2015 mortality and to those variables that showed a statistically-significant association with mortality, including the intervention as a dummy variable (presence vs. absence). All the analyses were carried out before and after weighting the UAs populations for their mean values during the summer 2015. With this procedure, ethical approval of the study was not required

## 3. Results

The two populations were made up of 6483 cases and 5724 controls, comparable for age and gender (Table 1).

From June–September 2015, 4720 older adults were contacted by the LLE program, by phone; 2963 received more than one phone call (2.22 phone calls per person on average) and 502 (10.6% of the total number) more than four phone calls. The discrepancy between the official residents and the citizens contacted by the program is mainly due to a real residence different from the official one and to the absence of the citizen due to spending holidays outside the city. The authors assume that this discrepancy should not affect cases and controls differently. The LLE program operators carried out 660 interventions other than phone calls for 143 older adults (3.0%): most of these interventions were “visiting the patients when admitted to the hospital” (111, 16.2% of the interventions other than phone calls) and “bringing food/medicine” at home (101, 15.3%). Other frequent interventions were “contact relatives/friends” (88 cases) and “search for a paid assistant” (73 cases): these interventions all together represent about 54% of the total number during the summer of 2015.

**Figure 2:**
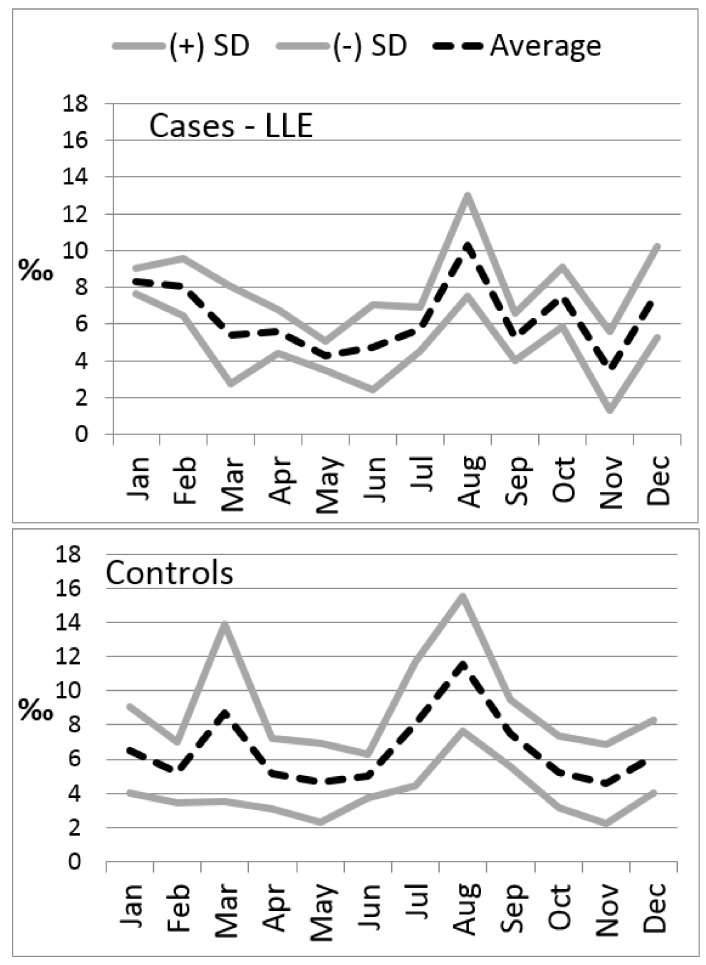
2015 average monthly death rate and standard deviation with and without an LLE program.

During the summer of 2015, 167 deaths were recorded in the UAs in which the LLE program was active, and 169 in those in which it was not, implying cumulative mortality rates of 25‰ (SD ± 1.4; Cl 95%: 23–29) and 29‰ (SD ± 6.7; Cl 95%: 17–43), respectively. The increase of deaths during the summer of 2015, compared to the summer of 2014, was more prominent in UAs in which the LLE program had not been implemented (+97.3% vs. +48.8%, Table 2). This was also the case for the average increase of deaths in comparison with the mortality recorded during the months before the summer of 2015 (35.4% vs. 6.6% from October 2014–May 2015), as well as for the average reduction during the months of October and November 2015 (−35.6% vs. −12.5%). The standard deviation between UAs of the summer of 2015’s average death rate was smaller in the UAs assisted by the LLE program than in the control UAs (Moses rank test <0.001—Figure 2). 

The difference of the summer and autumn percentage of cumulative mortalities between cases and controls (Table 2: Δ1 + Δ2 = 19.1% vs. 71.0%) can be taken as a measure of the resilience of the two populations during heat waves: the higher the resilience, the lower the cumulative variation.

**Figure 3:**
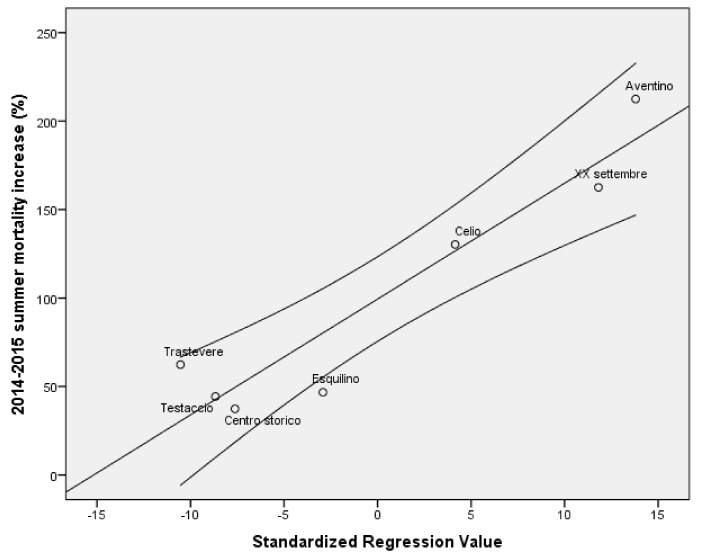
2014–2015 summer mortality increase (%); multivariable linear regression (R^2^ = 0.789).

If the cases had had the same death rate as the controls, the expected number of deaths during summer 2015 would have been 192, implying that 25 deaths were averted, a 13% reduction in mortality. The multivariable regression model (Figure 3, Table 3), weighted by the number of residents in each urban area, adjusted for the proportion of those over 90 and the pre-summer of 2015 mortality per urban areas, confirmed the statistical significance of the lower increase of mortality recorded in the urban areas served by the LLE program (β = 0.217; *p* < 0.001). Interestingly, the mean property tax valuation is associated with the overall mortality from May 2014–December 2016 (Pearson correlation = 0.829, *p* = 0.021), but is not associated with either the 2015 or the summer of 2015 mortality (Table S1). The gender balance, as the percentage of females in the population, did not show a statistically-significant difference according to the presence or not of the LLE program (62.0% in the LLE program population vs. 58.5% among the controls). Likewise, no association was observed between the share of females per UA and the summer of 2015 mortality. A marginal effect of gender on the overall mortality (cumulative mortality from June 2014–December 2016) can be observed, most probably as an effect of the higher percentage of females in the population. 

## 4. Discussion

An increase of mortality during the summer of 2015 was recorded in all the UAs, even if the percentage increase varied. The main differences observed among residents followed by the LLE program were the reduction of the expected mortality by about 13% during the summer, compared with residents in other UAs of central Rome, and the narrowing of the death rate variation among the UAs. The main factors affecting mortality were the age of residents in each UA, the pre-summer mortality and the implementation of the LLE program. There are many factors demonstrating the link connecting loneliness and a poor community network with higher mortality rates and vice versa [23,24,25,26,27,28]. In this case, we note a reduction in mortality, due to a program aimed at strengthening the individuals’ social relations, as well as the social capital at the community level. The program acts as a shock-absorber, able to reduce the mortality variation by implementing monitoring and targeted care activities at the community level; these are intensified during heat waves. In such periods, all the participants are called at least once, especially during the 2015 summer compared with other summers, due to the higher number of alerts. The impact of the LLE program on mortality is shown also by the reduction of mortality recorded in the months following the heat waves (Table 2), due to the harvesting effect (excess of mortality in previous months) that was reduced where the LLE program was operating. The smoothing of the mortality curve has already been acknowledged as the result of adaptation, through a period of time, to climatic changes (i.e., the increased frequency of heath waves) and of the prevention campaigns rolled out in Rome [13]. However, this was a historical comparison, which also included years with no severe heat waves. Any effect of the prevention campaign during extreme climatic events (like the heat waves that occurred in 2015) was not reported. In this case, the authors noted a smoothing of the mortality curve among residents benefitting from the LLE program, with a reduction of the mortality peak, as well as of the cumulative variation of mortality during the whole year, which could be seen as a measure of the population’s resilience to heat waves. Resilience can be defined as the capacity to cope with the consequences of disasters [33,34,35]: the resilience of a community is increased when the susceptibility to an extreme event is reduced, mainly by improving the residential environment, either by dealing with the social environment or with the physical environment. In this case, the smoothing of the mortality rate curve observed in the LLE program UAs can be considered a measure of the increase of resilience that results in a mitigation of the harvesting effect of summer heat waves. 

The mean property tax valuation as a proxy of the residents’ socio-economic status, was associated with mortality rates only if the whole period of observation was considered, while no association was observed with the 2015 mortality rates alone. This could be a further indirect demonstration of the positive impact of the LLE program: in fact, the provision of social services seems to break the link between higher mortality and lower socio-economic status reported by several authors [36,37,38]. The gender balance did not affect either the summer 2015 mortality or the 2015 increase of mortality, probably because the difference in the gender balance between cases and controls is small, close to 5% [13]. 

A longer period should be considered than the one more often allowed for in the analysis of heat wave events and their impact (mortality and morbidity) on populations of older adults. It was noted that the impact of cold snaps lasts up to three weeks after the event ends [39,40], while for heat waves, the results are generally not conclusive [41,42]. Since the causes of mortality amongst older adults are the same in either hot or cold weather (mainly cardiovascular and respiratory diseases), it is likely that the lag time could be similar in the two cases, at least for older adults and for similar durations of intense hot or cold spells. In fact, compared with younger people, the reaction to physical stress can be delayed in older adults, as it is to many other stimuli [43]. In Italy, the hospital length of stay is about 40% longer among those over 75 than among the 45–64-year-old age group, independently of the disease leading to admission [32]. It is likely that the onset, as well as the exacerbation of a disease caused by climatic events can have medium-/long-term consequences for older adults more frequently than is generally the case with younger people, especially when the interval between heat waves is short, in which case the negative effects caused by the first heat wave could still be active when the second begins. It is worth noticing that, during the summer of 2015, 23 alerts were recorded in Rome, which is on average one alert every six days. The increase of hospital admissions during hot days and heat waves has been reported [29,44]. However, there is also evidence of an undesirable correlation between temperature increases and hospitalization rates, or visit rates to emergency departments, by those over 65 (though there may well be a different temporal relationship from that between heat waves and morbidity in those over 75). The present study was not designed to take into account the impact of heat waves on hospitalization because it is based on data provided by the Municipality Statistical Office, which does not gather information about hospital care. Further ad hoc studies on the impact of social care on older adults’ use of hospital care are needed in order to shed more light onto this issue.

The results of this study should be treated with caution, because of its limitations: first of all, the data reported here are based on the assisted people’s administrative residence, which does not correspond to the place where they live. In fact, it is estimated that, in Rome, at least 10% of the people living in UAs are not resident, while the same percentage are resident, but living in a different place. Since mortality data are based on the official home address, some inaccuracies might occur, although the authors assume that they would affect all the UAs equally. A second limitation could be the lack of individual information on factors that can affect mortality, such as the presence of air conditioning in the house or the vacation time spent outside Rome during the summer of 2015. These conditions, which are likely to be enjoyed by the more affluent people among those under observation, can be taken into consideration by including in the model a proxy of the socio-economic status like the mean property tax valuation in a given UA. However, this variable did not affect the 2015 mortality, even if it showed a strong correlation with the overall 2014–2016 mortality. This could be an effect of the interventions, based on an assessment of multidimensional frailty, including socio-economic conditions, and focused on frail people, including those lacking socio-economic resources. The program probably reduces inequalities, providing support to those most in need, based on psycho-physical impairment and/or a lack of socio-economic resources. We do not know the immediate causes of deaths, even for those that occurred during heat waves, which are usually not so different from those under normal circumstances (mainly cardiovascular and/or respiratory diseases), so we cannot establish a direct relationship between heat waves and deaths that occurred during the summer. Again, the authors do not think this limitation should have different effects between different UAs.

## 5. Conclusions

In conclusion, the paper shows the impact of a community-based active monitoring program focused on strengthening individual relationship networks and the social capital of the community on the mortality of those over 75 during heat waves. The mitigation of mortality during the 2015 heat wave summer compared to non-heat wave summers is likely to be the outcome of the increase of resilience resulting from the implementation of the LLE program throughout the year and especially during climatic crises. Investments in social services associated with assessments of frailty carried out routinely in order to draft individual care plans could be a good strategy for improving people’s health and quality of life, at least during heat wave episodes.

## Figures and Tables

**Table 1:** Baseline parameters.

Urban Area	LLE Program	Population Average by Month during Summer 2015		Population Weight *	Females (%)	Mean Age (2015)	Share of Population over 90 (%)		Mean Property Tax Valuation (€) (2015)	
		Urban Areas	Total							
Centro Storico	No (Controls)	2837	5724	162.71	55.28	81.81	8.34	10.3	1977	1767
Aventino		1136		65.16	64.24	81.83	11.11		1636	
XX Settembre		1255		71.97	60.15	82.73	10.41		1787	
Celio		496		28.44	59.92	82.34	11.47		1669	
Trastevere	Yes (Cases)	1470	6483	84.27	65.35	82.41	8.58	9.0	1733	1592
Testaccio		1124		64.43	62.07	82.42	8.66		1370	
Esquilino		3889		223.02	59.56	82.57	9.92		1674	

* Calculated for the summer of 2015 population average per urban area = 1743.

**Table 2:** Average death rates and percentage differences of death rates by month and calendar year.

Urban Zone	Controls					Cases				Moses’ Rank Test ^2^
	Centro Storico	Aventino	XX Settembre	Celio	Mean (SD) ^1^	Trastevere	Testaccio	Esquilino	Mean (SD) ^1^	
Monthly average death rate (‰) October 2014–May 2015	5.75	5.45	4.72	8.34	6.0 (1.5)	6.17	5.86	6.67	6.2 (0.4)	NS
Monthly average death rate (‰) June–September 2014	4.17	2.82	3.65	3.50	3.7 (0.6)	3.66	4.93	4.38	4.3 (0.5)	<0.001
Monthly average death rate (‰) June–September 2015	5.73	8.80	9.57	8.06	7.3 (2.0)	5.95	7.12	6.43	6.4 (0.4)	<0.001
Δ1: June–September 2015 vs. June–September 2014 (%)	37.38	212.50	162.59	130.30	97.3 (73.1)	62.37	44.32	46.71	48.8 (6.8)	<0.001
Δ2: June–September 2015 vs. October 2014–May 2015 (%)	−0.41	61.62	102.96	−3.34	35.4 (43.0)	−3.50	21.47	−3.56	6.6 (6.7)	<0.001
Monthly average death rate (‰) October–November 2015	5.09	6.15	2.37	5.96	4.7 (1.6)	5.73	4.91	5.76	5.6 (0.4)	<0.001
Δ3: October–November 2015 vs. June–September 2015 (%)	−11.02	−30.08	−75.21	−26.00	−35.6 (30.1)	−3.67	−31.07	−10.46	−12.5 (10.5)	<0.001
Δ2−Δ3					71.0 (67.3)				19.1 (15.31)	NS

^1^ Weighted average for the population size in the summer of 2015; ^2^ The Moses’ rank test compares the dispersion of the values in the two groups, controls and cases.

**Table 3:** Multivariable linear regression: dependent variable: 2014–2015 summer mortality increase; weighted for the summer of 2015 mean population size, adjusted for the share of the over 90 population and the October 2014–May 2015 monthly mortality average; R^2^ = 0.789.

	Non-standardized Coefficient		Standardized Coefficient	*p*	95.0% Cl	
	B	Standard Error	Beta		Lower	Upper
(Constant)	−143.628	11.681		<0.001	−166.583	−120.673
October 2014–May 2015 monthly mortality average	−32.714	1.567	−0.431	<0.001	−35.793	−29.634
Share of the over 90 population	45.481	1.046	0.792	<0.001	43.426	47.537
LLE program (no vs. yes)	−24.372	2.370	−0.210	<0.001	−29.029	−19.714

## Supplementary Materials

The following are available online at http://www.mdpi.com/1660-4601/15/4/715/s1, File_data: a special dataset for this study, provided by the Statistical Office of the Municipality. Table S1: Variables tested against the mortality indicators (univariate linear regression).
