# Supplementary material for: Social Interventions to Prevent Heat-Related Mortality in the Older Adult in Rome, Italy: A Quasi-Experimental Study
Source: Int J Environ Res Public Health. 2018 Apr 11;15(4):715. doi: 10.3390/ijerph15040715 (PMC5923757; doi:10.3390/ijerph15040715)
Supplement: Supplementary file 1 [file ijerph-15-00715-s001.zip › GLrev_Supplemental materials_1.pdf]

Table S1. Variables tested against the mortality indicators (Univariate Linear Regression)

Urban areas		Cumulative death rate summer 2015	Increase of death (%) Jun-Sep 2014 – Jun-Sep 2015	Cumulative death rate 2015	Cumulative death rate jun 2014 – Dec 2016
Mean age Summer 2015	β	**0.777***	0.712	0.697	0.677
	p	**0.040**	0.073	0.082	0.095
Over-90 residents: share in summer 2015	β	**0.767**	**0.810**	**0.804**	0.503
	p	**0.044**	**0.027**	**0.029**	0.250
Percentage of females	β	0.385	0.282	0.420	0.742
	p	0.394	0.541	0.348	0.056
Property tax valuation	β	-0.196	-0.020	-0.404	**-0.829**
	p	0.673	0.965	0.369	**0.021**
Social discomfort index*	β	-0.519	-0.530	-0.388	0.080
	p	0.233	0.221	0.389	0.864
Housing discomfort index**	β	-0.557	-0.381	-0.144	-0.497
	p	0.194	0.399	0.759	0.256

* Combination of the following data calculated for the interested Urban Area: Unemployment rate, Employment rate, share of Youth (population aged less than 25) over the total population and Schooling rate

** Combination of the following data calculated for the interested Urban Area: share of residential buildings of the urban area of interest in a bad or mediocre state of conservation) over the Total residential buildings of the same urban area, adjusted for the weighting coefficient (0.168) which is the national percentage of residential buildings with "bad" or "mediocre" conservation status.
